# Risk factors and clinical characteristics of lung cancer in idiopathic pulmonary fibrosis: a retrospective cohort study

**DOI:** 10.1186/s12890-019-0905-8

**Published:** 2019-08-14

**Authors:** Hongseok Yoo, Byeong-Ho Jeong, Myung Jin Chung, Kyung Soo Lee, O. Jung Kwon, Man Pyo Chung

**Affiliations:** 1 Division of Pulmonary and Critical Care Medicine, Department of Medicine, Samsung Medical Center, Sungkyunkwan University School of Medicine, 81 Irwon-ro, Gangnamgu, Seoul, Republic of Korea; 2 Department of Radiology and Center for Imaging Science, Samsung Medical Center, Sungkyunkwan University School of Medicine, Seoul, Republic of Korea

**Keywords:** Idiopathic pulmonary fibrosis, Incidence, Lung cancer, Risk factors

## Abstract

**Background:**

Lung cancer is a common comorbidity of idiopathic pulmonary fibrosis (IPF) and has poor outcomes. The incidence and clinical factors related to development of lung cancer in idiopathic pulmonary fibrosis (IPF) are unclear. The aim of this study was to elucidate the cumulative incidence, risk factors, and clinical characteristics of lung cancer in IPF.

**Methods:**

In this retrospective study, we analyzed clinical data for 938 patients who were diagnosed with IPF without lung cancer between 1998 and 2013. Demographic, physiologic, radiographic, and histologic characteristics were reviewed. Cumulative incidence of lung cancer and survival were estimated by the Kaplan-Meier method. Risk factors of lung cancer development were determined by Cox proportional hazard analysis.

**Results:**

Among 938 IPF patients without lung cancer at initial diagnosis, lung cancer developed in 135 (14.5%) during the follow-up period. The cumulative incidences of lung cancer were 1.1% at 1 year, 8.7% at 3, 15.9% at 5, and 31.1% at 10 years. Risk factors of lung cancer were male gender, current smoking at IPF diagnosis, and rapid annual decline of 10% or more in forced vital capacity (FVC). Patients who developed lung cancer were mostly elderly men with smoking history. Squamous cell carcinoma followed by adenocarcinoma was the most common histologic type. Lung cancer was frequently located in areas abutting or within fibrosis. Survival was significantly worse in patients with lung cancer compared to patients with IPF alone.

**Conclusion:**

Lung cancer frequently developed in patients with IPF and was common in current-smoking men with rapid decline of FVC.

**Electronic supplementary material:**

The online version of this article (10.1186/s12890-019-0905-8) contains supplementary material, which is available to authorized users.

## Background

Idiopathic pulmonary fibrosis (IPF) is the most common type of idiopathic interstitial pneumonia, characterized by chronic progressive fibrosis of lung without etiologies [1]. Despite continuous efforts to develop therapeutic agents, no curative treatment exists for IPF. Thus, IPF is associated with poor prognosis, with median survival of 3 years following diagnosis [2]. Poor prognosis of IPF is attributable to the progressive nature of fibrosis, which is a unique characteristic of disease that leads to severe respiratory failure. However, recent studies demonstrated that complications of IPF such as acute exacerbation, coronary artery disease, pulmonary hypertension, gastroesophageal reflux disease may result in substantial mortality and morbidity [3–7].

One of the most fatal comorbidities of IPF is lung cancer with a reported prevalence of 4.4 to 48% in patients with IPF [8, 9]. Although the specific mechanism of lung cancer development in IPF is not fully understood, the increased incidence compared to patients without IPF has also been noted in large epidemiologic studies [9–11]. The significance of lung cancer in IPF lies not only in its high incidence but also in its impact on survival. Recent studies have shown that the comorbidity of lung cancer in IPF patients considerably reduces survival due to complications from treatment and from lung cancer itself [11–13]. Therefore, the need to identify predictive factors and clinical characteristics of lung cancer in IPF is essential for establishing screening protocols and diagnostic and therapeutic strategies. Despite its importance, few data on these subjects are available in the literature. Furthermore, most studies have limitations of a small number of investigated patients or analysis of a specific subset of patients; thus, they are not able to fully depict the nature of lung cancer in IPF patients [14–16]. In this study, we aimed to identify the cumulative incidence, risk factors, and clinical characteristics of lung cancer in patients during follow up of IPF.

## Methods

We conducted a cohort study based on a prospective registry of patients with IPF at Samsung Medical Center (a 1961-bed, university-affiliated, tertiary referral hospital in Seoul, Republic of Korea). In our hospital, all consecutive patients diagnosed with interstitial lung disease in the interstitial lung disease clinic are prospectively registered in an interstitial lung disease database since January, 1998. From the database, we identified 1360 patients who were diagnosed with IPF based on the diagnostic criteria of the American Thoracic Society and European Respiratory Society [1] between January 1998 and April 2013. Records of these patients were reviewed for possible inclusion in analysis. Excluded were 235 patients who were followed at our hospital for less than 6 months and 10 who did not have sufficient data for analysis. In addition, 172 patients diagnosed with lung cancer concurrently or within 6 months of IPF diagnosis and 5 patients transferred to our hospital with treated lung cancer were excluded. Finally, 938 patients without lung cancer at the time of IPF diagnosis and with fully available data were considered eligible for analysis. The Institutional Review Board of Samsung Medical Center approved the collection, analysis, and publication of the data and informed consent was waived due to the retrospective nature of the study.

The following clinical data were obtained from the medical records: age, gender, comorbidities, symptoms at the time of lung cancer diagnosis, use of corticosteroid or azathioprine for IPF treatment, results of pulmonary function tests at initial and 1 year after IPF diagnosis and at the time of lung cancer diagnosis, radiologic findings at the time of IPF and lung cancer diagnosis, histologic type and stage of lung cancer, and mortality. Chest radiography and chest computed tomography (CT) images were thoroughly reviewed by two authors (MJC and KSL). CT fibrosis score was defined as percentage of lung affected by fibrosis that included reticulation/honeycombing and CT emphysema score was defined as percentage of lung affected by emphysema [17, 18]. Percentages were rounded to the nearest 5%. Rapid decline of forced vital capacity (FVC) and diffusing capacity for carbon monoxide (DLco) were defined as annual decline of FVC of 10% or more and DLco of 15% or more, respectively [19, 20].

Categorical variables are reported as numbers (percentages). Continuous variables with normal distribution are reported as mean with standard deviation while variables with nonnormal distribution are reported as median with interquartile ranges (IQR, 25th to 75th percentiles). Categorical variables were compared using chi-square test and continuous variables using either independent *t*-test or Mann Whitney *U* test according to normalness of distribution. The annual decline of FVC and DLco was calculated using the results of pulmonary function tests at the time of IPF diagnosis and at 1 year after the IPF diagnosis. Regarding the patients in whom lung cancer developed within 1 year of IPF diagnosis, the last pulmonary function test undertaken at the time without the evidence of lung cancer was adopted for estimation of annual decline. We estimated the cumulative incidence of lung cancer with the Kaplan-Meier method. Cox proportional hazard model using backward stepwise selection method was used to identify independent predictive factors for lung cancer development with careful selection of variables after univariate regression analysis. The cumulative incidence of lung cancer according to the predictive factors identified by Cox proportional hazard model was estimated by Kaplan-Meier method. The statistical significance was determined by log-rank test. Kaplan-Meier estimation was used to determine the survival curves for patients with and without lung cancer, which were then compared using the log-rank test.

All tests were two-sided and a *P* value < 0.05 was considered significant. Data were analyzed using IBM SPSS Statistics 20 (IBM, Chicago, IL, USA).

## Results

**Fig. 1:**
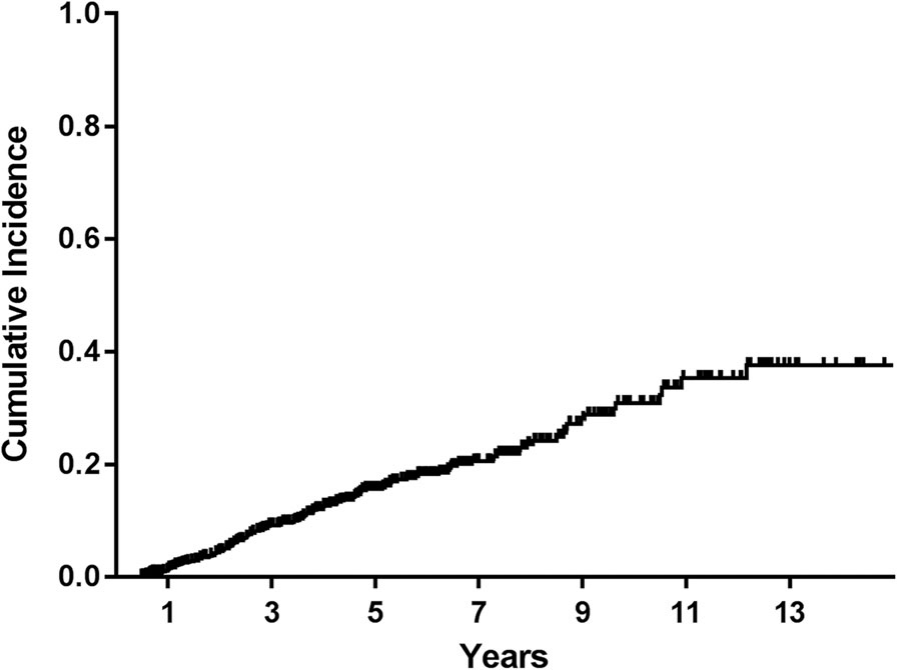
Cumulative incidence of lung cancer development in patients with idiopathic pulmonary fibrosis using Kaplan-Meier curve. Cumulative incidences were 1.1% at 1 year, 8.7% at 3, 15.9% at 5, and 31.1% at 10 years

The baseline characteristics of 938 patients at the time of IPF diagnosis are summarized in Table 1. Mean age at IPF diagnosis was 65.6 years. Most patients were male (79.3%) and current (23.5%) or ex-smokers (49.3%). Mean duration of follow up was 4.5 years. The median interval between pulmonary function tests was 13 (IQR, 12–20) months. Among the 938 patients, lung cancer developed in 135. The cumulative incidences of lung cancer were 1.1% at 1 year, 8.7% at 3, 15.9% at 5, and 31.1% at 10 years (Fig. 1). The incidence density rate was 32.6/1000 person-years.

**Table 1:** Clinical characteristics of study patients. (*N* = 938)

	Total (*N* = 938)	IPF with lung cancer (*n* = 135)	IPF without lung cancer (*n* = 803)	*P* value
Age, years	65.6 ± 8.1	65.2 ± 7.2	65.7 ± 8.2	0.479
Gender, male	744 (79.3)	128 (94.8)	616 (76.7)	< 0.001
Smoking				
Current	220 (23.5)	60 (44.4)	160 (19.9)	< 0.001
Ex-smoker	462 (49.3)	74 (54.8)	390 (48.6)	
Never smoker	256 (27.3)	1 (0.8)	253 (31.5)	
FVC, %	83.0 ± 18.6	89.5 ± 14.8	81.9 ± 19.0	< 0.001
FVC < 80%	316/818 (38.6)	24/115 (20.9)	292/703 (41.5)	< 0.001
DLco, %	71.3 ± 20.9	73.2 ± 20.3	71.0 ± 21.0	0.290
DLco < 80%	475/724 (65.6)	67/105 (63.8)	408/619 (65.9)	0.675
FEV1/FVC < 70%	101/795 (12.7)	25/111 (22.5)	76/684 (11.1)	0.001
Decline of FVC ≥ 10%/year^a^	148/691 (21.4)	18/92 (19.6)	130/599 (21.7)	0.642
Decline of DLco ≥15%/year^a^	132/571 (23.1)	12/81 (14.8)	120/490 (24.5)	0.056
Use of azathioprine^b^	96 (10.2)	11 (8.1)	85 (10.6)	0.387
Use of steroid^b^	123 (13.1)	13 (9.6)	110 (13.7)	0.195
Follow-up duration, years	4.5 ± 3.1	3.8 ± 2.6	4.6 ± 3.2	0.002

*DLco* diffusing capacity for carbon monoxide, *FEV1* forced expiratory volume in 1 s, *FVC* forced vital capacity, *IPF* idiopathic pulmonary fibrosis

^a^ Annual decline of FVC and DLco was calculated using the results of pulmonary function tests at the time of IPF diagnosis and at one year after the IPF diagnosis

^b^ Use of medication and duration of follow-up for patients who developed lung cancer are applicable to period from IPF diagnosis to lung cancer development

**Fig. 2:**
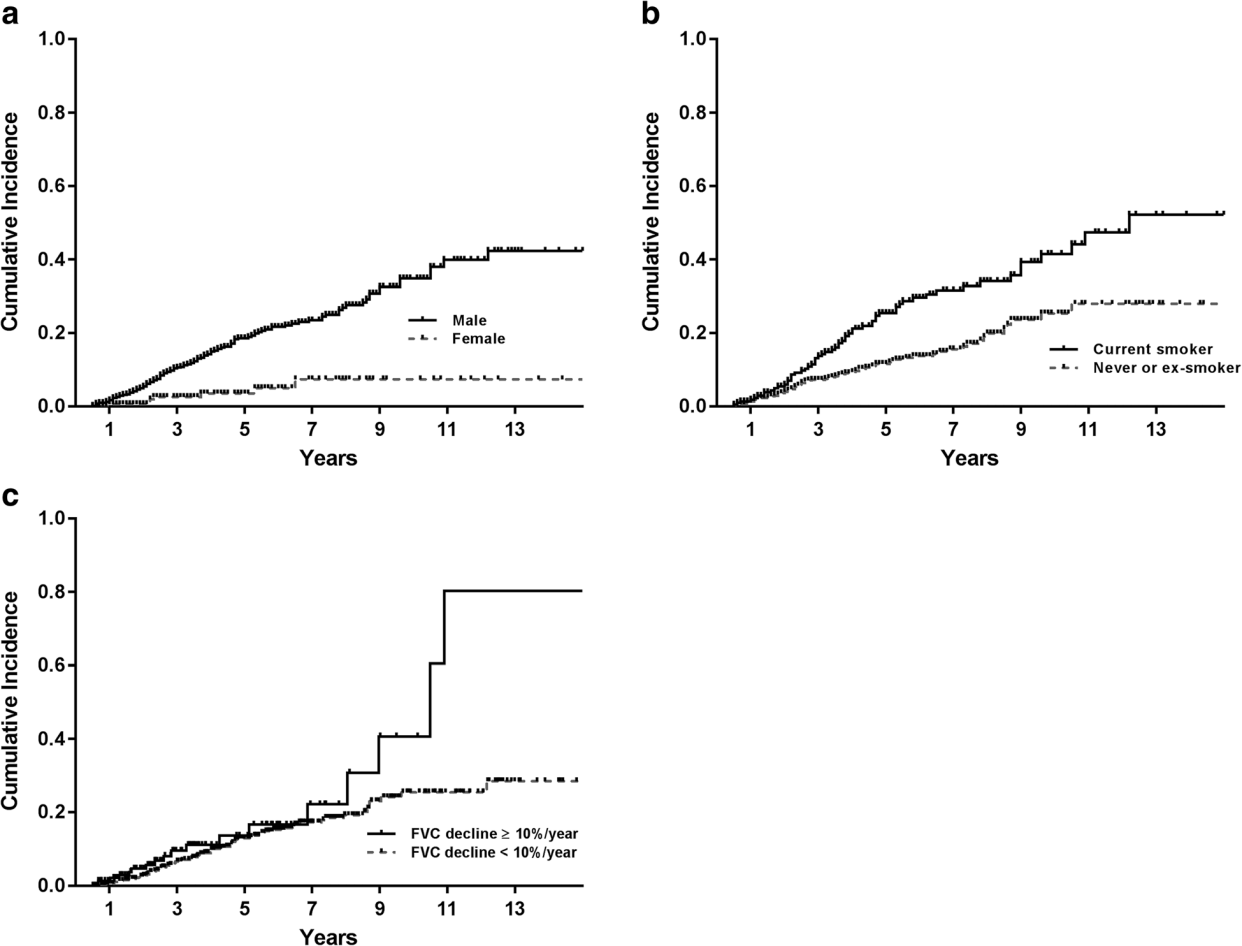
Cumulative incidence of lung cancer development according to (**a**) gender (*P* < 0.001, log-rank test), **b** smoking status at diagnosis of idiopathic pulmonary fibrosis (*P* < 0.001, log-rank test), and (**c**) annual decline of 10% or more in forced vital capacity (*P* = 0.0563, log-rank test) using Kaplan-Meier curve

Patients with lung cancer were more commonly male and current or ex-smokers at IPF diagnosis. In addition, they had better FVC and were more likely to have airflow limitation (Forced expiratory volume in 1 s/FVC < 70%) (Table 1). Univariate Cox regression analysis for proportional risk on baseline characteristics showed male gender, current smoking, better FVC, and airflow limitation at IPF diagnosis were associated with lung cancer development. Although not statistically significant, rapid annual decline in FVC was associated with a tendency toward lung cancer development (Table 2). Multivariate Cox regression analysis showed male gender, current smoking, and rapid decline in FVC were independently associated with lung cancer development (Table 2, Fig. 2). When subgroup analysis on current and ex-smokers was performed, pack-year of smoking and rapid decline of FVC were significantly associated with lung cancer development (Additional file 1: Table S1, Additional file 1: Figure S1).

**Table 2:** Univariate and multivariate Cox regression analysis for factors associated with lung cancer development

	Crude hazard ratio (95% CI)	*P* value	Adjusted hazard ratio (95% CI)	*P* value
Age, years	1.017 (0.996–1.038)	0.122		
Gender, male	4.648 (2.171–9.949)	< 0.001	15.956 (2.204–115.496)	0.006
Current smoking	2.055 (1.460–2.891)	< 0.001	1.864 (1.185–2.931)	0.007
FVC < 80%	0.594 (0.377–0.934)	0.024		
DLco < 80%	1.162 (0.777–1.738)	0.464		
FEV1/FVC < 70%	1.653 (1.057–2.585)	0.027		
Decline of FVC ≥ 10%/year	1.653 (0.981–2.782)	0.059	1.857 (1.014–3.400)	0.045
Decline of DLco ≥15%/year	0.954 (0.515–1.769)	0.882		
Use of azathioprine	0.720 (0.388–1.334)	0.296		
Use of steroid	0.843 (0.475–1.493)	0.558		

*CI* confidence interval, *DLco* diffusing capacity, *FEV1* forced expiratory volume in 1 s, *FVC* forced vital capacity

Detailed characteristics of 135 patients with lung cancer are in Table 3. Mean age at lung cancer diagnosis was 69.0 years. Most patients were male (94.8%) and were current or ex-smokers. Median interval between IPF diagnosis and lung cancer was 38 months (IQR, 20–67). Median FVC was 84% (IQR, 71–93) and DLco was 62% (IQR, 50–77) at lung cancer diagnosis.

**Table 3:** Characteristic of patients who developed lung cancer at the time of lung cancer diagnosis (*n* = 135)

	No. (%) or mean ± SD or median (IQR)
Age, years	
At the time of IPF diagnosis	65.2 ± 7.2
At the time of lung cancer diagnosis	69.0 ± 7.2
Gender, male	128 (94.8)
Smoking	
Current smoker	26 (19.3)
Ex-smoker	108 (80.0)
Pack-year	41.7 ± 13.7
Comorbidities	96 (71.1)
Diabetes	41 (30.4)
Chronic heart disease	33 (24.4)
Previous history of tuberculosis	29 (21.5)
Other malignancy	16 (11.9)
Cerebrovascular disease	8 (5.9)
Chronic liver disease	6 (4.4)
Chronic kidney disease	5 (3.7)
Symptoms	
Development of new symptoms	58 (43.0)
Change of symptoms	24 (17.8)
Pulmonary function test at lung cancer diagnosis	
FVC, % (*n* = 125)	84 (71–93)
DLco, % (*n* = 113)	62 (50–77)
TLC, % (*n* = 99)	83 (73–91)
6-min walk test, distance, meter (*n* = 38)	420 (360–474)
6-min walk test, lowest saturation, % (*n* = 38)	89 (82–93)

*DLco* diffusing capacity, *FVC* forced vital capacity, *IPF* idiopathic pulmonary fibrosis, *IQR* interquartile range, *SD* standard deviation, *TLC* total lung capacity

For histologic types of lung cancer, squamous cell carcinoma was the most common (32.6%) followed by adenocarcinoma (28.1%). Twenty-seven (20.0%) patients were diagnosed with small cell carcinoma. For 17 (12.6%) of patients who were diagnosed with non-small cell carcinoma, specific cell type could not be determined due to insufficient amount of specimen. Pathology review of one patient showed both squamous and small cells. Details on histopathologic types and stages of lung cancer are available on supplement. (Additional file 1: Tables S2, S3, and S4).

Chest radiographs and CT scans at lung cancer diagnosis were available for review in all patients. (Table 4) Visible lung lesions on chest radiograph were observed in 104 (77.0%) patients. Lung cancer frequently developed in regions abutting fibrosis (29.6%) or within fibrosis (44.4%). Median CT fibrosis score was 25 (IQR, 15–38) and median CT emphysema score was 10 (IQR, 0–20). The time interval from most recent chest CT without lung cancer to chest CT at lung cancer diagnosis was 23 months (IQR, 11–43). The number of patients with chest CT interval of 1 year or less was 35 (26.7%). The stages of lung cancer did not differ between patients whose interval of chest CT scans was 1 year or less and more than 1 year (*P* for trend = 0.141). (Additional file 1: Table S5) However, the proportion of stage I lung cancer was higher in patients whose interval of chest CT scans was 1 year or less (38.9% vs. 21.2%, *P* = 0.038).

**Table 4:** Radiographic findings of lung cancer (*n* = 135)

	No. (%) or median (IQR)
Detectable lesion on chest radiography	104 (77.0)
Morphology of main lesion	
Round or oval	82 (60.7)
Irregular	43 (31.9)
Stellate	5 (3.7)
Band-like	2 (1.5)
No parenchymal lesion	3 (2.2)
Size of main lesion, mm	33 (21–50)
Location of main lesion	
Right upper lobe	26 (19.3)
Right middle lobe	10 (7.4)
Right lower lobe	42 (31.1)
Left upper lobe	26 (19.3)
Left lower lobe	28 (20.7)
Relation to IPF	
Abutting fibrosis	40 (29.6)
Within fibrosis	60 (44.4)
Abutting ground glass opacity	3 (2.2)
Normal lung parenchyma	29 (21.5)
CT fibrosis score	25 (15–38)
CT emphysema score	10 (0–20)

*CT* computed tomography, *IPF* idiopathic pulmonary fibrosis, *IQR* interquartile range

Survival of patients with and without lung cancer was analyzed. The median survival of patients with lung cancer was 3.4 years compared to 9.8 years in patients without lung cancer. The difference was statistically significant (*P* < 0.001). (Additional file 1: Figure S2).

## Discussion

Although several studies demonstrated increased prevalence of lung cancer in IPF, only a few studies thoroughly evaluated the cumulative incidence of lung cancer. Ozawa et al. reported 1-, 5-, and 10-year cumulative incidences of lung cancer of 3.3, 15.4, and 54.7% after retrospectively evaluating 103 patients with IPF at their institute [21]. A study by Tomassetti et al. found that lung cancer developed in 23 (30%) of 181 patients with IPF [12]. When calculated only for patients who developed lung cancer, 1- and 3-year cumulative incidences were 41 and 82%, respectively, suggesting most lung cancer in IPF develops within 3 years [12]. Most recently, Yoon et al. identified 31 (2.8%) lung cancer out of 1108 patients with IPF by reviewing their interstitial lung disease registry. Although exact cumulative incidences are not reported in the article, they noted that the incidence of lung cancer was increased in the first 2 years of IPF diagnosis. [11] In addition, the incidence was 3.34 times higher in IPF compared to that of age-adjusted general population. Finally, in a recent systematic review, the estimated adjusted incidence rate ratio from 2 large cohort studies was reported to be 6.42 after adjustment for age, smoking, and gender. [22] The high incidence density rate of 32.6/1000 person-years and 10-year cumulative incidence of 31.1% in our study is in line with previous studies that IPF is frequently associated with lung cancer. Of note, the difference in our study lies in exclusion of patients who were diagnosed with IPF and lung cancer simultaneously and the relatively large number of patients. The study by Tomassetti et al. not only included patients who were diagnosed with lung cancer during follow-up of IPF but also patients who were diagnosed with lung cancer and IPF concurrently which constituted 30% of the 23 lung cancer patients. The study by Yoon et al. also included patients who were diagnosed with lung cancer and IPF at the same time which consisted approximately 20% of lung cancer patients. This inclusion could be the reason for the relatively high early cumulative incidence observed in their studies. Thus, our study may provide more insight on cumulative incidence of lung cancer development in patients during follow up of IPF, which increased persistently over time.

Considering the high incidence of lung cancer and its impact on survival of patients with IPF, understanding the predictive factors of lung cancer development is the first step for clinicians to establish surveillance protocols. Older age at IPF diagnosis, male gender, smoking, and emphysema have been proposed as possible risk factors for lung cancer development in previous studies [12, 21, 23, 24]. Nevertheless, the risk factors of lung cancer still remain unclear because of the small number of evaluated patients, resulting in insufficient statistical power; analysis of characteristics at the time of lung cancer diagnosis; and inclusion of patients concomitantly diagnosed with lung cancer and IPF. In our analysis, male gender, current smoking at the time of IPF diagnosis, and rapid annual decline of FVC were determined to be predictive factors of future lung cancer occurrence.

One of the most noteworthy finding in our study was that rapid decline of FVC was independently associated with lung cancer development. To the best of our knowledge, this is the first study to evaluate and demonstrate the relationship between changes in pulmonary function and risk of lung cancer development in patients with IPF. Although the reason for this association is unclear, the suggested mechanism of frequent lung cancer development in IPF may explain this observation. A longstanding hypothesis is that shared pathogenesis of tissue damage and abnormal repair, which are key processes in IPF and lung cancer development, is the reason that patients with IPF are vulnerable to cancer occurrence [25]. The finding that lung cancer arises commonly abutting or within fibrosis may support this hypothesis. Recent advances in molecular techniques also offer genetic- and epigenetic-level evidence that abnormal DNA methylation and histone modification leading to abnormal gene expression or aberrant activation of signaling pathways are shared by both IPF and cancer [26]. Thus, patients with rapid decline in FVC, which reflects the active and progressive status of IPF, may be more prone to lung cancer development, considering the common pathogenesis of IPF and lung cancer. However, further studies are warranted to confirm this hypothesis.

The results of our study on demographic, clinical, histologic, and radiologic characteristics of patients with IPF and lung cancer did not differ from previous studies. Most patients were older men with a current or ex-smoking history [12, 13, 21]. Median time from diagnosis of IPF to lung cancer was 38 months, similar to 30 months in a study by Tomassetti et al. [12]. Median FVC and DLco were slightly lower than in previous studies. This difference may be because we included only patients with a diagnosis of lung cancer during follow-up for IPF, thus excluding patients diagnosed incidentally with pulmonary fibrosis who might have been in relatively early stage IPF [7, 13, 21]. Previous studies demonstrated that squamous cell carcinoma is the most common histologic type encountered in IPF [10, 21, 26]. Our data concurs, with 32.6% of squamous cell carcinoma. Radiological findings were in line with previous studies with round or ovoid masses frequently observed abutting or within fibrosis [27, 28]. Although both upper and lower lobe predilections have been described [27, 29], lesions in lower lobes were more frequent in our study.

One of the radiologic findings that requires an attention in our study is that the proportion of stage I lung cancer was statistically higher in patients whose interval of chest CT scans was 1 year or less. Despite the frequency of lung cancer and its detrimental impact on prognosis in patients with IPF, currently there is no screening protocol available for these patients [1]. Although the guidelines recommend lung cancer screening with annual low dose chest CT scan in high risk patients [30, 31], the benefit of this recommendation has not been validated in patients with IPF. The result of our study may suggest a possibility that annual or shorter-term CT screening may also be beneficial in early detection of lung cancer in patients with IPF. Nevertheless, the retrospective observational nature of this study and various reasons which may have initiated short-term CT scan follow-up limit the interpretation of the results. In addition, considering the high rate of treatment related complications and possible non-eligibility for curative treatment due to poor lung function in patients with IPF [13, 32], the survival benefit must be scrutinized as well. Further studies are necessary to determine the benefit of the lung cancer surveillance with chest CT scan and optimal duration of follow-up for patients with IPF.

Significant reduction in survival for patients with lung cancer and IPF compared to that of patients with IPF alone was portrayed in our study. Although Ozawa et al. [21] noted no difference in survival between IPF patients with and without lung cancer, most recent studies consistently reported worse survival in patients with lung cancer [11, 12]. This detrimental impact of lung cancer on IPF is known to be largely attributable to progression of lung cancer or treatment-related complications. [12] The high incidence and effects of lung cancer on patients with IPF emphasizes the importance of establishing a surveillance protocol for early diagnosis as well as defining effective and safe treatment modalities for those patients.

Potential limitations should be acknowledged to fully appreciate the results of our study. First, given the observational nature, there is always the possibility that selection bias of confounding might have influenced our findings. In addition, our study was conducted at a single referral institution with an interstitial lung disease clinic, which may limit its generalizability to other settings. Second, we were not able to determine the association between use of antifibrotic agents and risk of lung cancer development. A recent study reported that treatment with pirfenidone was associated with reduced incidence of lung cancer in patients with IPF. [33] However, since our study consisted of patients diagnosed between 1998 and 2005, only a small proportion of the study patients received pirfenidone or nintedanib which limited us from further analysis.

## Conclusion

In summary, lung cancer frequently and persistently developed in patients with IPF. Current smokers who were men with rapidly declining FVC were more prone to lung cancer occurrence. Squamous cell carcinoma was the most common histologic type.

## Additional files

Additional file 1 **Table S1**. Univariate and multivariate Cox regression analysis for factors associated with lung cancer development in current and ex-smokers. (*n* = 684). **Table S2**. Histopathologic types of lung cancer (*n* = 135). **Table S3**. TNM stages of lung cancer (n = 135). **Table S4**. Stages of non-small cell carcinoma and small cell carcinoma (n = 135). **Table S5**. Stages of lung cancer in patients with chest CT interval of 1 year or less and more than 1 year (n = 135). **Figure S1**. Cumulative incidence of lung cancer development according to pack-years of smoking using Kaplan-Meier curve in current and ex-smokers. (n = 684) (*P* < 0.001, log-rank test). **Figure S2**. Kaplan-Meier survival analysis comparing IPF patients with and without lung cancer. (*P* < 0.001, log-rank test) (DOCX 293 kb)

## Data Availability

Data and material are available on reasonable request.

## Abbreviations

CPFE: Combined pulmonary fibrosis and emphysema
CT: Computed tomography
DLco: Diffusion capacity for carbon monoxide
FVC: Forced vital capacity
IPF: Idiopathic pulmonary fibrosis
IQR: Interquartile range
