# Supplementary material for: Risk factors and clinical characteristics of lung cancer in idiopathic pulmonary fibrosis: a retrospective cohort study
Source: BMC Pulm Med. 2019 Aug 14;19:149. doi: 10.1186/s12890-019-0905-8 (PMC6693185; doi:10.1186/s12890-019-0905-8)

**Risk factors and clinical characteristics of lung cancer in idiopathic pulmonary fibrosis: A retrospective cohort study**

table S1. Univariate and multivariate Cox regression analysis for factors associated with lung cancer development in current and ex-smokers. (n=684)

|  | Crude hazard ratio (95% CI) | *P* value | Adjusted hazard ratio (95% CI) | *P* value |
| --- | --- | --- | --- | --- |
| Age, years | 1.020 (0.999 – 1.041) | 0.064 |  |  |
| Gender, male | 0.877 (0.358 – 2.148) | 0.773 |  |  |
| Current smoking | 1.316 (0.934 – 1.854) | 0.117 |  |  |
| Pack-year | 1.024 (1.014 – 1.033) | <0.001 | 1.029 (1.016 – 1.043) | <0.001 |
| FVC < 80% | 0.695 (0.442 – 1.094) | 0.116 |  |  |
| DLco < 80% | 0.217 (0.813 – 1.822) | 0.339 |  |  |
| FEV1/FVC < 70% | 1.250 (0.799 – 1.956) | 0.328 |  |  |
| Decline of FVC ≥ 10%/year | 1.871 (1.114 – 3.143) | 0.018 | 1.988 (1.088 – 3.634) | 0.025 |
| Decline of DLco ≥ 15%/year | 0.909 (0.491 – 1.681) | 0.760 |  |  |
| Use of azathioprine | 0.863 (0.466 – 1.601) | 0.863 |  |  |
| Use of steroid | 0.975 (0.550 – 1.730) | 0.932 |  |  |

CI, confidence interval; DLco, diffusing capacity; FEV1, forced expiratory volume in 1 second; FVC, forced vital capacity;

table S2. Histopathologic types of lung cancer (n=135)

|  | No. (%) |
| --- | --- |
| Non-small cell carcinoma  Squamous cell  Adenocarcinoma  Non-small cell^*^  Large cell  Pleomorphic  Squamous and adenocarcinoma  Squamous (90%) and small cell (10%)  Small cell carcinoma | 108 (80.0)  44 (32.6)  38 (28.1)  17 (12.6)  5 (3.7)  2 (1.5)  1 (0.7)  1 (0.7)  27 (20.0) |

^*^ Specific cell type could not be differentiated due to small amount of specimen.

table S3. TNM stages of lung cancer (n=135)

|  | No (%) |
| --- | --- |
| Stage IA  Stage IB  Stage IIA  Stage IIB  Stage IIIA  Stage IIIB  Stage IV | 21 (15.6)  14 (10.4)  5 (3.7)  8 (5.9)  31 (23.0)  14 (10.4)  42 (31.1) |

table S4. Stages of non-small cell carcinoma and small cell carcinoma (n=135)

|  | No (%) |
| --- | --- |
| Non-small cell carcinoma  Stage IA  Stage IB  Stage IIA  Stage IIB  Stage IIIA  Stage IIIB  Stage IV | 18 (16.7)  12 (11.1)  4 (3.7)  7 (6.5)  23 (21.3)  10 (9.3)  34 (31.5) |
| Small cell carcinoma  Limited disease  Extensive disease | 19 (70.4)  8 (29.6) |

table S5. Stages of lung cancer in patients with chest CT interval of 1 year or less and more than 1 year (n=135)

|  | Chest CT interval of 1 year or less (n=36, 26.7%) | Chest CT interval of more than 1 year (n=99, 73.3%) | P value |
| --- | --- | --- | --- |
| Stage IA  Stage IB  Stage IIA  Stage IIB  Stage IIIA  Stage IIIB  Stage IV | 9 (25.0)  5 (13.9)  1 (2.8)  2 (5.6)  5 (13.9)  2 (5.6)  12 (33.3) | 12 (12.1)  9 (9.1)  4 (4.0)  6 (6.1)  26 (26.3)  12 (12.1)  30 (30.3) | 0.141 |

figure S1. Cumulative incidence of lung cancer development according to pack-years of smoking using Kaplan-Meier curve in current and ex-smokers. (n=684) (*P* < 0.001, log-rank test)


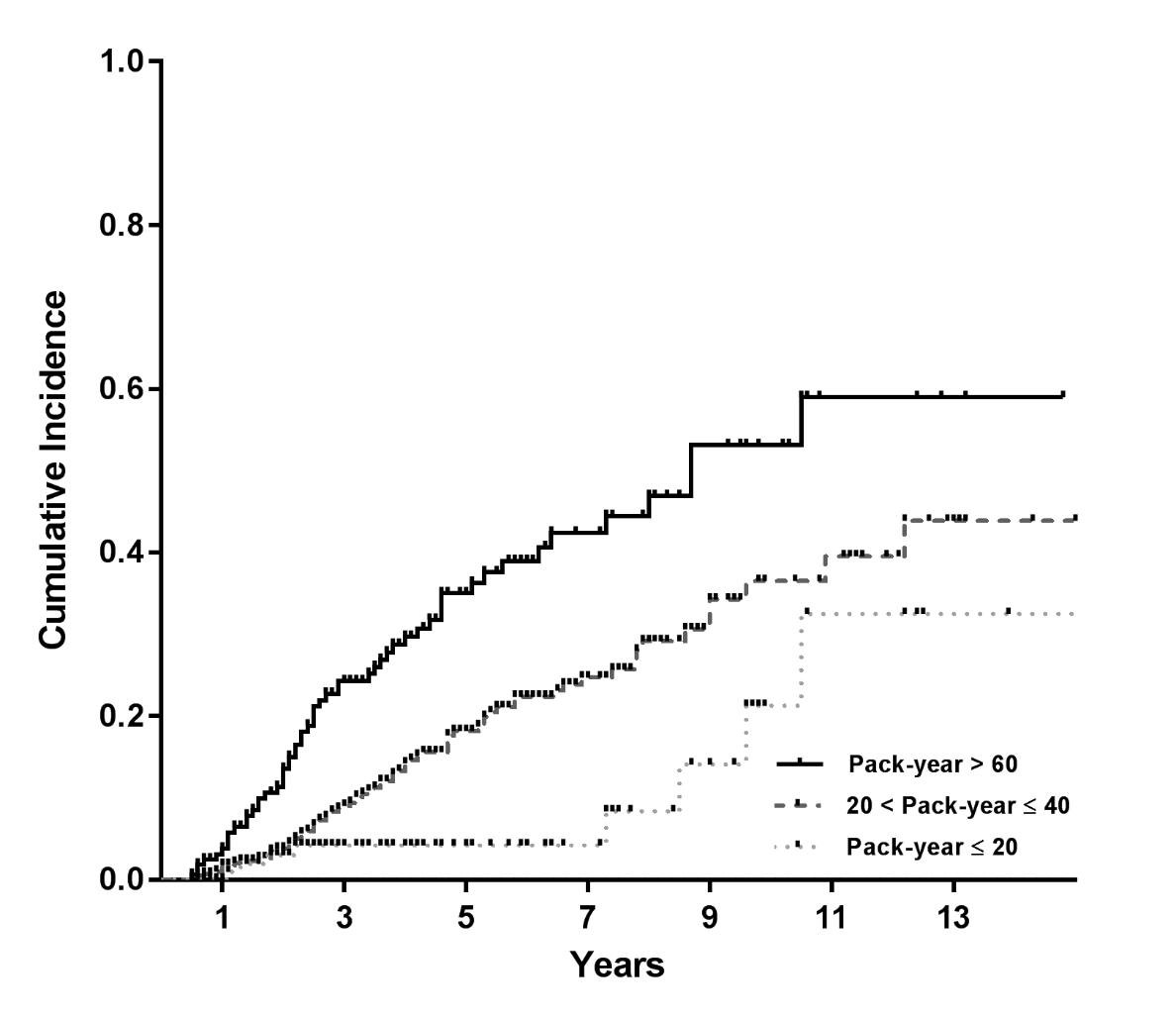


figure S2. Kaplan-Meier survival analysis comparing IPF patients with and without lung cancer. (*P* < 0.001, log-rank test)


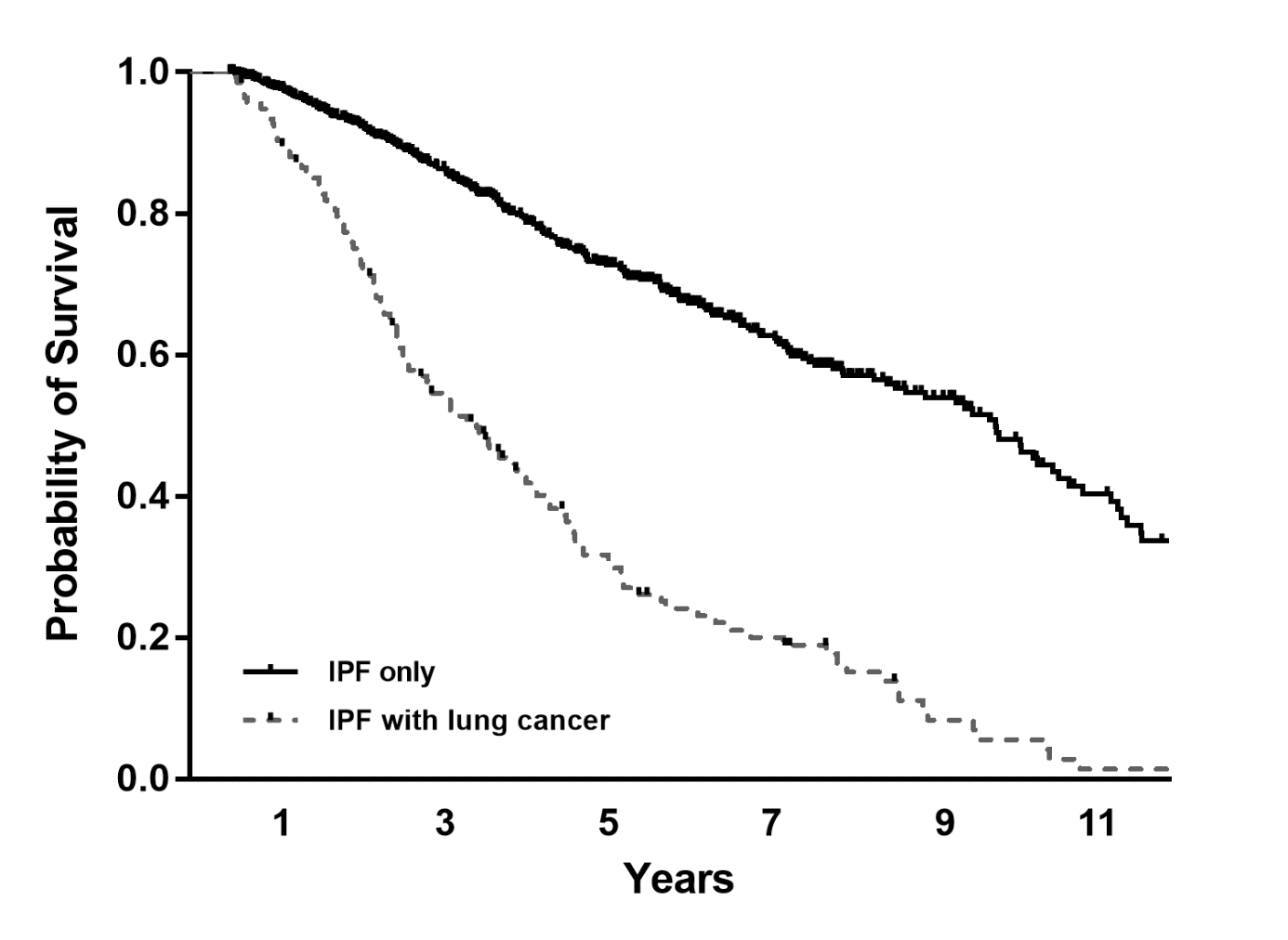

Supplement: Supplementary file 1 — Table S1. Univariate and multivariate Cox regression analysis for factors associated with lung cancer development in current and ex-smokers. (n = 684). Table S2. Histopathologic types of lung cancer (n = 135). Table S3. TNM stages of lung cancer (n = 135). Table S4. Stages of non-small cell carcinoma and small cell carcinoma (n = 135). Table S5. Stages of lung cancer in patients with chest CT interval of 1 year or less and more than 1 year (n = 135). Figure S1. Cumulative incidence of lung cancer development according to pack-years of smoking using Kaplan-Meier curve in current and ex-smokers. (n = 684) (P < 0.001, log-rank test). Figure S2. Kaplan-Meier survival analysis comparing IPF patients with and without lung cancer. (P < 0.001, log-rank test) (DOCX 293 kb) [file 12890_2019_905_MOESM1_ESM.docx]
